# Biological Characteristics and Antimicrobial Activity of Endophytic* Streptomyces* sp. TQR12-4 Isolated from Elite* Citrus nobilis* Cultivar Ham Yen of Vietnam

**DOI:** 10.1155/2016/7207818

**Published:** 2016-10-04

**Authors:** Phan Thi Hong-Thao, Nguyen Vu Mai-Linh, Nguyen Thi Hong-Lien, Nguyen Van Hieu

**Affiliations:** Institute of Biotechnology, Vietnam Academy of Science and Technology, 18 Hoang Quoc Viet Street, Cau Giay, Ha Noi, Vietnam

## Abstract

Ham Yen orange (*Citrus nobilis* Lour) is the highly valuable commercial fruit of Vietnam. With the blooming of fruit production and farming area, this specialty crop is facing threats from several serious diseases; therefore the search for new effective biocontrollers is required to prevent the existing excessive use of fertilizers and plant protection chemicals. Endophytic actinomycetes are of great scientific interest due to their high potential of application in agriculture and pharmaceutical research. In this work, endophytic actinomycetes were isolated from a native orange species of Northeast mountainous province Tuyen Quang. Among 49 isolates obtained, the isolate TQR12-4 strongly inhibited test pathogens* Colletotrichum truncatum*,* Geotrichum candidum*,* Fusarium oxysporum*, and* F. udum*. This isolate gave comparatively high biomass yields on different substrates, for example, carboxy methyl cellulose, starch, protein, and chitin, within a wide range of temperature from 15 to 45°C and pH from 4 to 10. Sequence analysis of 16S rDNA gene showed that TQR12-4 shared 99% similarity to* Streptomyces prasinopilosus*; however, it slightly differed from the latter in spore morphology and hence was named as* Streptomyces* sp. TQR12-4. A thermostable antifungal substance of nonpeptide nature produced by* Streptomyces* sp. TQR12-4 had MIC against* Fusarium udum* of 100 *μ*g/mL and 400 *μ*g/mL respective to extract fractions *X*_4_ and *X*_5_.

## 1. Introduction

Ham Yen orange tree has been domesticated since 1890s by Muong minority people in Ham Yen district of mountainous province Tuyen Quang of Northeast Vietnam. This species produces the most nutritious and economically valuable fruit with current growing area of 4,900 hectares and yield of about 43,000 tons by 2015 and brings about the significant economic benefit for the local growers. Like other citrus species, Ham Yen orange plants are attacked by many diseases such as Tristeza, greening, citrus canker caused by various phytopathogens resulting in decreasing the yield and quality of the crop. Application of synthetic fertilizers and other chemicals to control the disease is not only very much effective but also hazardous to consumers and environment. Therefore, the search for effective disease biocontrollers from other organisms, especially microorganism, is necessary to reduce agrochemical inputs.

Endophytes are defined as the microorganisms that colonize in living plant tissues (roots, branches, and leaves) without causing visible harm to the host plant and can be isolated from surface disinfected plant tissues or extracted from inside the plant [1]. According to Kandpal et al. (2012), they can be found in almost all plant species studied to date and are recognised to be important for the development of host plant through microbe-host interaction [2]. While endophytes receive nutrients (mainly carbohydrates) from the host plant, the latter obtains compounds or secondary metabolites and antibiotics produced by endophytes to enhance the plant growth and to gain protection from pathogens [3–7].

Among studied endophytes,* Streptomyces* species, which are isolated from a diversity of plant species, are one particular group of interest because of their ability to produce antibiotics as well as other bioactive secondary metabolites [1, 8–16]. Of about 23,000 bioactive secondary metabolites discovered in endophytic microorganisms, approximately 10,000 substances were produced by endophytic actinomycetes and 7,600 substances were found in* Streptomyces* genus [17, 18].

Currently, there are only a limited number of reports on the presence of endophytes in citrus plants. According to Araújo et al. (2002),* Physoderma citri* was the first endophytic fungus found in healthy* Citrus sinensis*. After that, a number of bacteria including members of* Achromobacter*,* Acinetobacter*,* Alcaligenes*,* Arthrobacter*,* Bacillus*,* Burkholderia*,* Citrobacter*,* Corynebacterium*,* Enterobacter*, and* Pseudomonas *have been isolated from xylem of lemon roots [19]. Recently, Douanla-Meli et al. (2013) reported on the isolation of other endophytic fungi in citrus plants [20]. Shutsrirung et al. (2013) studied the biodiversity of endophytic actinomycetes in mandarin grown in Thailand and their production of growth regulating substances [21]. Kandpal et al. (2012) were interested in the antimicrobial activity of endophytic actinomycetes in* Citrus aurantifolia* grown in different regions of India [2].

In Vietnam, research on the endophytic microorganisms is at early stages. Nevertheless, the search for new substances from endophytic microorganisms is drawing a great attention. In this study, endophytic streptomycetes of Ham Yen orange were isolated from roots, branches, and leaves. Biological characteristics, identification, and substances production of the most active isolate TQR12-4 were extensively investigated. The results showed that TQR12-4 exhibited remarkably strong activity against four phytopathogenic fungi,* Colletotrichum truncatum*,* Geotrichum candidum*,* Fusarium oxysporum*, and* Fusarium udum*, and two Gram-positive bacteria,* Bacillus subtilis* and* Staphylococcus aureus*.

## 2. Materials and Methods

### 2.1. Materials

Root, branch, and leaf samples were collected from seven-year-old orange trees grown in two mountainous villages, Deo-Ang and Hoc-Trai, of Ham Yen district, Tuyen Quang province. Test (indicator) microorganisms are used in this study including* Colletotrichum truncatum *VSVD 14 (causing anthracnose of citrus fruits),* Geotrichum candidum* VSVD 1 (sour rot of citrus fruits and vegetables),* Fusarium oxysporum *FO 5 (fusarium wilt),* Fusarium udum *VSVD 4 (wilt), and* B. subtilis* ATCC 6633 received from the Culture Collection of Soil Microbiology Laboratory, Inst. of Biology, Vietnam Academy of Science and Technology, and* Staphylococcus aureus* ATCC 25922 and* Pseudomonas aeruginosa* ATCC 10145 obtained from the National Institute of Drug Quality Control. Primers for amplification of 16S rDNA were purchased from Invitrogen (Hong Kong).

### 2.2. Isolation of Endophytic Actinomycetes

Plant samples were surface-sterilized as described by Shutsrirung et al. (2013) [21]. Then, the samples were homogenized in a mortar with about 2–5 mL of sterile water. The liquid was collected and spread on the screening medium containing 2.0 g/L K_2_HPO_4_, 0.02 g/L CaCO_3_, 2.0 g/L KNO_3_, 0.01 g/L FeSO_4_·7H_2_O, 2.0 g/L NaCl, 0.05 g/L MgSO_4_·H_2_O, 1.0 g/L humic acid, 10 mL of 0.2 N NaOH, and 18.0 g/L agar-agar, pH 7.0. To prevent the growth of bacteria and fungi, nystatin and nalidixic acid were added to the medium to the final concentrations of 100 mg/L and 10 mg/L, respectively. The inoculated plates were incubated at 28°C. Typical actinomycete colonies were selected from day 15 to day 60 and purified by consecutive streaking on ISP medium #2 containing 10 g/L glucose, 5 g/L meat extract, 4 g/L malt extract, and 18 g/L agar-agar, pH 7.0.

### 2.3. Morphological and Cultural Characterization

Morphological and cultural characterization of the isolates was studied according to the protocols of the International Streptomyces Project (ISP) [22–24]. The spores were examined under Scanning Electron Microscope FESEM S4800.

Qualitative evaluation of enzyme activity was done by plate assay method. Carboxy methyl cellulose, chitin, oat spelt xylan, casein, and starch were used to detect respective enzymes: cellulase, chitinase, xylanase, protease, and amylase [25]. The activity of an enzyme was exhibited as a clear zone (degraded substrate) around the colony. For detection of amylase and xylanase, before observation the plates were flooded with iodine and Congo Red solutions, respectively.

Suitable growth temperatures and pH and salinity tolerances of the isolates were determined on the ISP medium #2. The experiments were carried out at different temperatures from 15 to 50°C, pH from 3 to 9, and NaCl concentrations from 3 to 7%. The results were recorded after 14 days.

### 2.4. DNA Extraction and 16S rRNA Gene Amplification

Cultivation of the isolate was performed according to Ishikawa (2000) [26]. Genomic DNA was isolated using NucleoSpin® Tissue extraction kit (Macherey-Nagel, Germany) according to the manufacturer's instructions. The 16S rRNA genes were amplified using primers 27f (5′-TAACACATGCAAGTCGAACG-3′) and 1492R (5′-GGTTACCTTGTTACGACTT-3′) [27] and genomic DNA. The thermal cycle for PCR was described by Shutsrirung et al. (2013) [21]. The 16S rDNA nucleotide sequence of isolates was aligned with relevant known sequences from GenBank using http://www.ncbi.nlm.nih.gov/BLAST to obtain reference sequences. Phylogenetic analysis was done by CLC DNA workbench 6.6.

### 2.5. Screening for Antibacterial Activity

The screening was performed using agar diffusion method. Three test bacteria,* B. subtilis* ATCC 6633,* S. aureus* ATCC25922, and* P. aeruginosa* ATCC10145, were grown separately in LB broth for 24 hours. A 100 *μ*L of a test bacterial culture was thoroughly spread on each of three LB agar plates. Agar blocks (Φ 5 mm) of actinomyces isolates previously grown for 7 days on ISP medium #2 were cut by a cork borer and placed on the LB plates inoculated with test bacterium. After overnight incubation at 37°C, growth inhibition was evaluated by measuring diameters of the halos surrounding the agar blocks.

### 2.6. Screening for Antifungal Activity

This assay was conducted by agar diffusion or coculture method as described by Intra et al. (2011) [28], with some modifications. Initially, the test fungus was grown on Hansen agar for 10 days. The conidial suspension was prepared in saline solution to the density of 10^6^ CFU/mL. Each Hansen agar plate was spread with 100 *μ*L of conidial suspension. Six agar plugs (Φ 5 mm) were removed from every plate and replaced by six actinomycete plugs, each containing a single isolate colony previously grown on ISP medium #2. The results were recorded after 3 days.

Alternatively, the test fungus and actinomycete isolate were inoculated by parallel streaking on Hansen agar at the distance of 3 cm from each other (coculture). The plates were incubated at 28°C and inhibition of mycelial growth was measured after 7 days.

### 2.7. Thermal and pH Stability of the Crude Antifungal Substance

The antifungal substance of selected isolate TQR12-4 was produced in medium AH4 containing 10 g/L glucose, 10 g/L soybean meal, 5 g/L NaCl, and 1 g/L CaCO_3_, pH 7. The cultivation lasted for 120 hours at 28°C. The crude antifungal substance was extracted from the entire culture with ethyl acetate [28]. After evaporation of solvent in vacuum, the crude substance was dissolved in 5% methanol for evaluation of thermal and pH stability using agar well diffusion assay against test fungi,* F. oxysporum*,* F. udum*, and* G. candidum*.

The solution of crude substance was treated at temperatures of 28, 40, 50, 60, 70, 80, and 100°C during 30, 60, 90, and 120 minutes for each temperature.

The pH of crude antifungal substance was adjusted to values of 3, 4, 5, 6, 7, 8, and 9 and kept at 28°C for 60 minutes.

### 2.8. Recovery and Bioassay of Antifungal Substance

The antifungal substance was extracted from 120-hour submerged culture using ethyl acetate as solvent. The solvent was evaporated in vacuum, and the solid crude substance was dissolved in methanol for separation on silica gel column using gradient elution of CH_2_Cl_2_/CH_3_OH (50/1–1/1, v/v). Antifungal activity of recovered fractions was examined against* F. udum* using disk diffusion method. The dried fractions were redissolved in methanol to the concentration of 10 mg/mL. Sterile Whatman filter paper disks (Φ 5 mm) were loaded, each with 50 *μ*L (0.5 mg) of a fraction solution, while the controls were loaded with only methanol. The loaded disks were left to dry at room temperature. Hansen agar plates were inoculated with the test fungus as described above. Triple test was performed, where 3 disks, each loaded with one fraction, and a control were placed on a plate. The diameters of inhibition zones were measured after incubation for 2 days at 28°C.

### 2.9. Determination of Minimum Inhibitory Concentration

The minimum inhibitory concentration (MIC) of fractions *X*_4_ and *X*_5_ was determined according to Narayana et al. (2007) [29] and Andrews (2001) [30], with some modifications. A commercial antifungal agent, carbendazim, was assayed in parallel for comparison. The test fungus,* F. udum* VSVD 4, was grown on Hansen agar medium for 10 days; then conidia were collected and suspended in saline solution to the density of 10^6^ CFU/mL.

Fractions *X*_4_ and *X*_5_ and carbendazim were first dissolved in methanol solution of 5% and then diluted with Hansen broth to concentrations of 0.84 ÷ 200 *μ*g/mL, 1.68 ÷ 400 *μ*g/mL, and 3.125 ÷ 100 *μ*g/mL, respectively.

In a test tube, equal volumes of 100 *μ*L of the conidial suspension and *X*_4_ or *X*_5_ or carbendazim were mixed together. The control tube contained only Hansen broth and conidial suspension of* F. udum*. The tubes were incubated aerobically for 72 hrs at 25°C. MIC values were recorded with no visible fungal growth at the lowest concentration.

## 3. Results and Discussion

### 3.1. Isolation of Endophytic Actinomycetes

**Figure 1:**
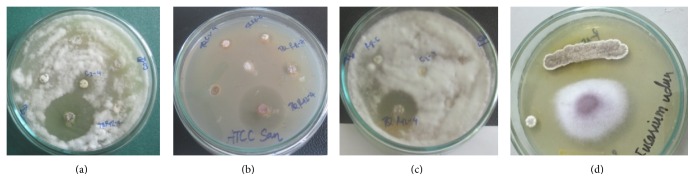
Screening of endophytic actinomycetes for antifungal and antibacterial activity. Test strains:* C. truncatum* VSVD 14 (a),* S. aureus* ATCC 25922 (b),* F. oxysporum *FO5 (c), and* F. udum *VSVD 4 (d).

Forty-nine endophyte actinomycetes were isolated from Ham Yen orange plant samples. Of them, 18 isolates (36.7%) showed antimicrobial activity against test organisms. Particularly, 5 isolates inhibited growth of 6 to 7 test organisms, and 7 isolates inhibited 3 to 4 test organisms. The isolate TQR12-4 exhibited the highest activity against 4 test pathogenic fungi,* Colletotrichum truncatum*,* Geotrichum candidum*,* Fusarium oxysporum*, and* Fusarium udum*. The culture broth of this isolate inhibited the growth of Gram-positive* S. aureus* ATCC 25922 and* B. subtilis *ATCC 6633 but did not inhibit Gram-negative* P. aeruginosa *ATCC 25932 (Table 1 and Figure 1).

### 3.2. Biological Characteristics of TQR12-4 Isolate

**Figure 2:**
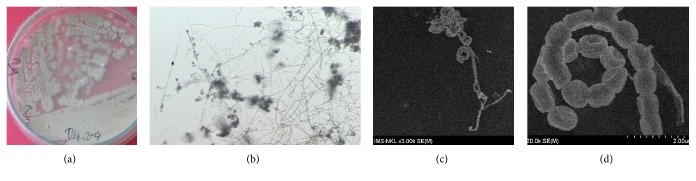
Morphology of isolate TQR12-4 grown on ISP medium #4 (7-day culture): colonies (a); light microscope image (×40); mycelia and spore chains (b); scanning electron micrograph: spore chain (c); and spores (d). Bar = 2 *μ*m.

The colony surface was powdery and curled. Aerial and substrate mycelia were grey to light brown or light yellow to brownish yellow on media ISP2, ISP3, ISP4, ISP5, and ISP7. Soluble yellow pigment was observed on media ISP2 and ISP3. Its spiral spore chains bore from several to about 30 smooth spores/chain (Figure 2).

In culture, isolate TQR12-4 can grow at temperature of 15 ÷ 45°C and pH of 4 ÷ 10. However, the most favorable temperature was 28°C and pH was 6.0 ÷ 7.0. Its salinity tolerance was 3% NaCl. The isolate degraded chitin, protein, starch, guaiacol, carboxymethyl cellulose, and assimilated carbon sources: D-glucose, D-xylose, D-mannitol, D-fructose, and D-cellulose. It showed week growth on L-arabinose, D-sucrose, and D-rhamnose and no growth on D-raffinose.

**Figure 3:**
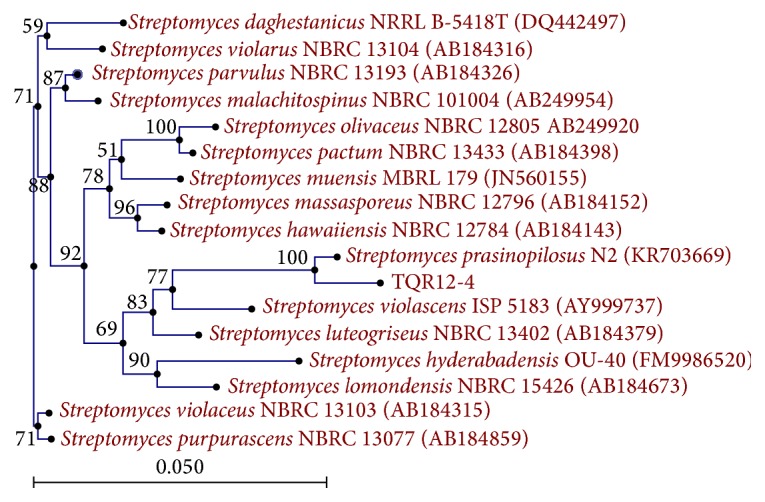
Neighbor-joining tree showing the phylogenetic relationships based on 16S rRNA gene sequence of the isolate TQR12-4 and closest species.

The obtained 16S rDNA nucleotide sequence (1361 bp) was registered at the National Center for Biotechnology Information (NCBI) GenBank database under accession number KX257804. It was compared with relative sequences available on GenBank database using BLAST tool on NCBI. It shared high similarity (99%) with the sequences of* Streptomyces *sp., for example,* Streptomyces prasinopilosus* N2 (KR703669) and* Streptomyces hawaiiensis* NBRC12784 (AB184143) (Figure 3). Despite such high similarity with* Streptomyces prasinopilosus*, there was still a slight difference in spore morphology between them. Therefore, our isolate was named as* Streptomyces* sp. TQR12-4.

### 3.3. Thermal and pH Stability of the Antifungal Substance

The crude extract of antifungal substance from* Streptomyces* sp. TQR12-4 showed activity against all three test fungi over a wide range of pH values from 3 to 9. The most suitable pH was 6 ÷ 7; otherwise, the activity slightly declined towards acidic or basic pH. Compared with the highest activity at pH 7.0, after 1-hour treatment at extreme pH of 3 and 9, the activity was reduced by 36 and 26%, respectively (Figures 5 and 6).

**Figure 4:**
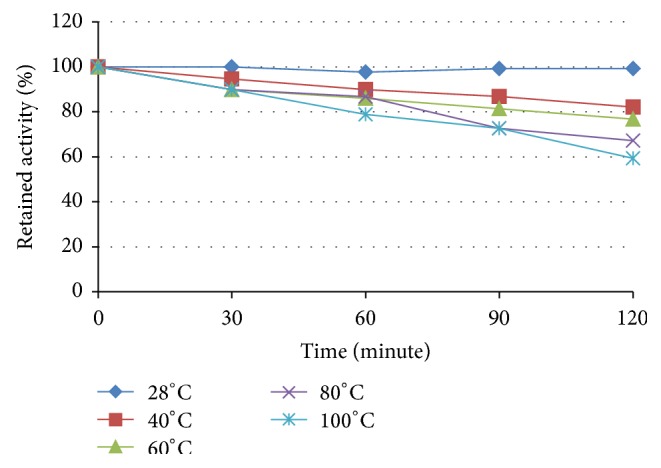
Thermal stability of the antifungal substance against* F. oxysporum*.

**Figure 5:**
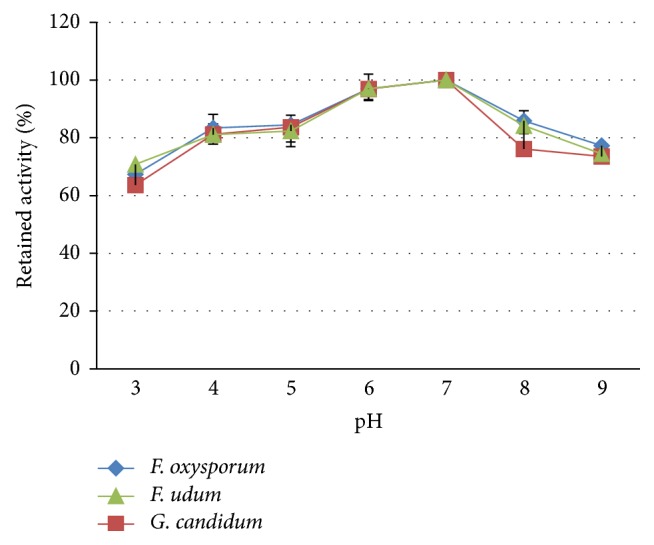
Stability to pH of the antifungal substance.

**Figure 6:**
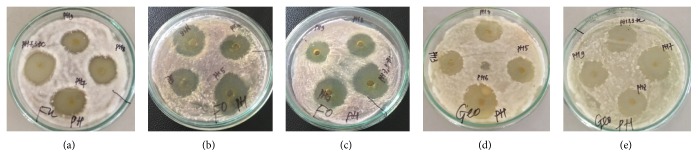
Well diffusion assay for stability to pH of the antifungal substance. (a)* F. udum*, (b, c)* F. oxysporum*, and (d, e)* G. candidum*.

Treatment at different temperatures showed relatively high thermal stability of the crude substance. Assuming that the antifungal activity at 28°C was 100%, incubation for 120 minutes at 60 and 100°C resulted in its decrease to 77% and 60%, respectively. Because of high similarity in responses of all three test fungi, only data for* F. oxysporum* were presented in Figure 4.

In the study by Prapagdee et al. (2008), the culture filtrates of* Streptomyces hygroscopicus* were treated at 100°C for 45 minutes and then tested against* Colletotrichum gloeosporioides* and* Sclerotium rolfsii*. The findings showed that the antifungal activity of the exponential culture was mainly assigned to heat labile proteins such as extracellular hydrolytic enzymes, while the activity of stationary culture was exhibited by more stable compounds of another nature [31]. Carvalho and Van Der Sand (2016) reported that the centrifuged culture broth of an endophytic actinomycete R18(6) might be exposed to 80°C for 30 minutes without losing much of antibacterial activity. However, that extract did not stand treatment at 100°C, as its activity started to drop after 3 minutes and was completely lost after 30 minutes. The author suggested the presence of peptide bonds in the molecule of that substance [32]. Since the substance produced by our strain* Streptomyces* sp. TQR12-4 was extracted with ethyl acetate and then subjected to treatment at 100°C for 120 minutes but still retained 60% of its activity, it may be of nonpeptide nature.

### 3.4. Bioassay of the Antifungal Substance

The crude antifungal substance was separated on silica gel column giving five fractions labelled as *X*_1_ to *X*_5_. The dry weights of recovered fractions were 300, 45, 36, 78, and 46 mg, respectively.

**Figure 7:**
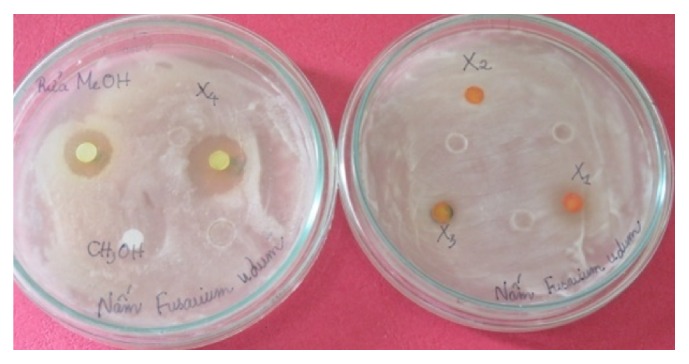
Activity against* F. udum* of recovered fractions. Faction *X*_5_ is to the left of *X*_4_ on the left plate.

The bioassay of all five fractions which was performed by well diffusion method revealed that fractions *X*_1_, *X*_2_, and *X*_3_ did not inhibit test fungal growth. Meanwhile, fractions *X*_4_ and *X*_5_ gave clear inhibition zones of 17 and 15 mm in diameter, respectively (Figure 7). The MICs against* F. udum* of fractions *X*_4_ and *X*_5_ and carbendazim were 100, 400, and 50 *μ*g/mL, respectively.

The antimicrobial activity of a certain substance depends on the strain-producer and test microorganisms. In a study by Kim et al. (2013), antifungal niphimycin from* Streptomyces* sp. KP6107 inhibited strains of genera* Alternaria*,* Aspergillus*,* Colletotrichum*,* Cercospora*,* Cylindrocarpon*,* Fusarium*, and* Rhizoctonia* with MIC of 8 ÷ 64 *μ*g/mL [33]. In assays with vegetative mycelia, the MICs of a bioactive compound, 3-phenylpropionic acid (3-PPA), from* Streptomyces albidoflavus* ANU 6277 against* Aspergillus flavus*,* A. niger*,* Fusarium oxysporum*,* F. udum*,* Penicillium citrinum*, and* Candida albicans* were of 10 ÷ 100 *μ*g/mL [29]. For spores of* F. udum*, the MICs of carbendazim, 3-PPA, and tricyclazole were 50, 100, and 500 *μ*g/mL, respectively. Our substance, produced by* Streptomyces* sp. TQR12-4, showed similar activity against* F. udum*, that is, MIC of 100 *μ*g/mL, and MIC of carbendazim was of 50 *μ*g/mL. However, further study must be carried out to understand the chemical nature of this substance and more of its properties.

As mentioned above, endophytic actinomycetes are reported to play an important role in the growth, physiology, and health of the host plant via their metabolic activity and mechanisms such as antibiosis, niche competition, and induction of host systemic resistance. However, the relationship between the endophytic microorganism and the host remains insufficiently explained.

Taechowisan et al. (2005) [34] studied antifungal substances produced by endophytic* Streptomyces aureofaciens* CMUAc130 isolated from the root tissue of* Zingiber officinale*. The substances were identified as 5,7-dimethoxy-4-p-methoxylphenylcoumarin and 5,7-dimethoxy-4-phenylcoumarin, which inhibited the phytopathogenic fungi* Colletotrichum musae* and* Fusarium oxysporum*. Interestingly, as summarized by the authors, 5,7-dimethoxy-4-p-methoxylphenylcoumarin can be extracted from a number of plants but not from the members of Zingiberaceae, which in turn host the streptomycete producing this substance. In our opinion, this fact may be a suggestion to study on the origin of certain antimicrobial compounds found in plants.

## 4. Conclusions

A streptomycete TQR12-4 isolated from Ham Yen orange specialty of Northeast Vietnam showed strong activity against 2 Gram-positive bacteria,* B. subtilis* and* S. aureus*, and 4 phytopathogenic fungi,* C. truncatum*,* G. candidum*,* F. oxysporum*, and* F. udum*. Biological and taxonomical characteristics and sequence analysis of 16S rDNA gene indicated that this isolate belongs to genus* Streptomyces*. It shares 99% similarity with* Streptomyces prasinopilosus* but has a slight difference in spore morphology and hence was named as* Streptomyces *sp. TQR12-4. The crude antimicrobial substance from this strain was comparatively stable over wide ranges of pH (3 to 9) and temperatures (40 to 100°C). Separation on silica gel gave five fractions, two of which (*X*_4_ and *X*_5_) inhibited the growth of* F. udum* at MIC of 100 and 400 *μ*g/mL, respectively. Studied properties of this substance indicate its remarkable advantages in production by microbiological fermentation and application in agricultural practice. To achieve these goals, further research is required to understand the chemical nature of this substance and more of its antimicrobial spectrum and production.

## Figures and Tables

**Table 1:** Antimicrobial activity of the endophytic actinomycete isolate TQR12-4.

Test organisms	Zone of inhibition (*D*, mm)	
	Agar block	Well diffusion
*S. aureus* ATCC 25922	26 ± 0,42	25 ± 1,00
*B. subtilis *ATCC 6633	25 ± 0,50	25 ± 0,42
*P. aeruginosa *ATCC 25932	0	0
*G. candidum *VSVD 1	28 ± 0,81	19 ± 0,35
*F. oxysporum *FO5	25 ± 0,50	17 ± 0,76
*F. udum *VSVD 4	26 ± 0,32	18 ± 0,58
*C. truncatum* VSVD 14	29 ± 0,81	20 ± 0,29

The results were recorded in 7-day culture on ISP medium #2.
